# Supplementary material for: Dopaminergic mechanism underlying reward-encoding of punishment omission during reversal learning in Drosophila
Source: Nat Commun. 2021 Feb 18;12:1115. doi: 10.1038/s41467-021-21388-w (PMC7893153; doi:10.1038/s41467-021-21388-w)
Supplement: Supplementary file 1 — Supplementary Information [file 41467_2021_21388_MOESM1_ESM.pdf]

Supplementary Information

Dopaminergic mechanism underlying reward-encoding of punishment omission during reversal learning in *Drosophila*

Li Yan McCurdy, Preeti Sareen, Pasha A. Davoudian, Michael N. Nitabach

This PDF file includes:

Supplementary Figures and Figure Legends 1-9
Supplementary Table 1: genotypes of flies used
Statistics and sample sizes for data in main figures
Statistics and sample sizes for data in supplementary figures

Supplementary Figure 1

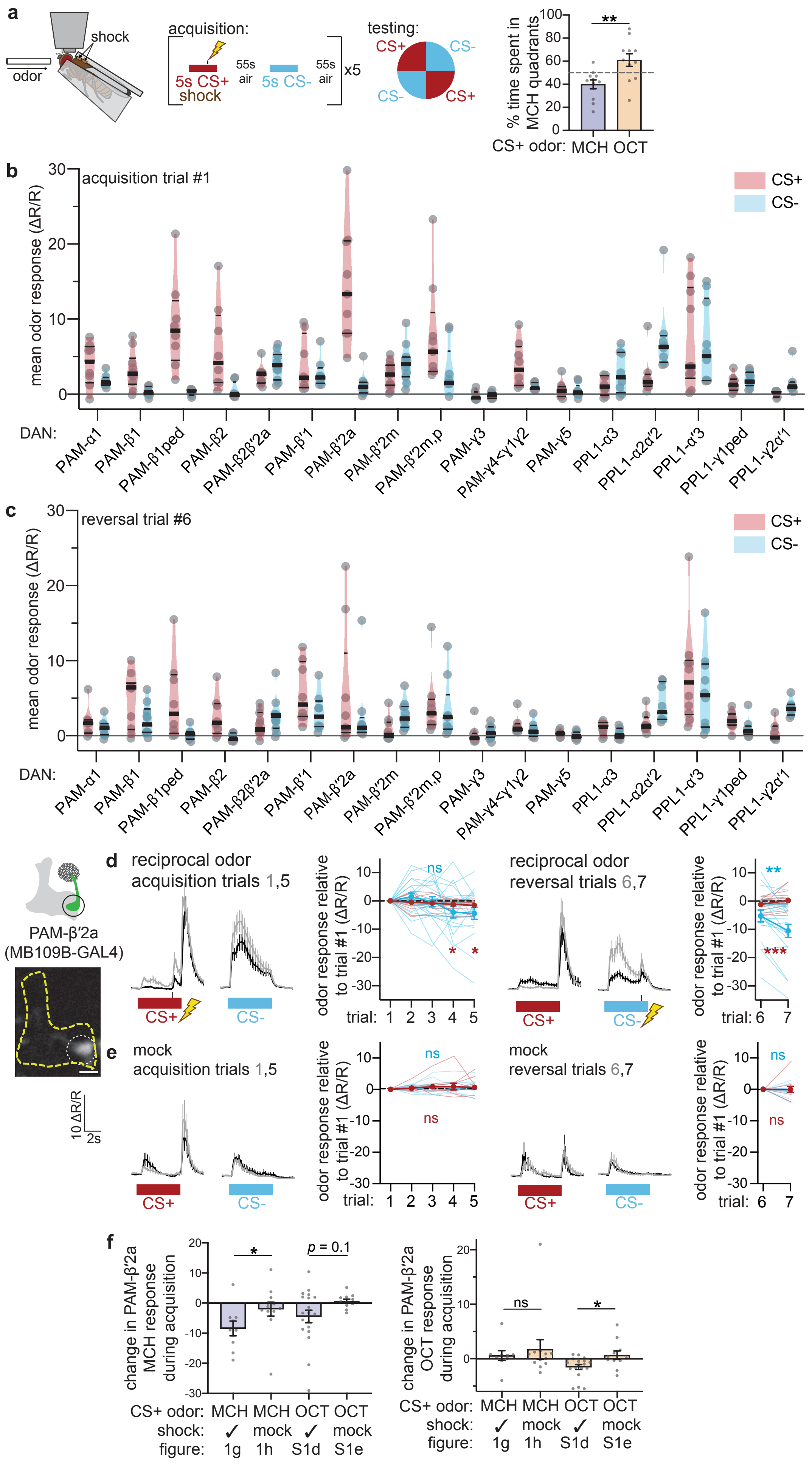

Supplementary Figure 1: Related to Figure 1

(**a**) Validation of experimental setup. Each fly is conditioned (acquisition only) in the imaging setup, without head-fixing or cuticle dissection. It is then placed in the quadrant arena and the percentage of time spent in CS+ versus CS- quadrants is quantified. Flies conditioned to associate the odor 3-octanol (OCT, CS+) with electric shock spend more time in MCH (CS-) quadrants, compared to flies conditioned with MCH as CS+. n = 11 flies per condition. Statistical comparison is by unpaired t-test.

(**b**) DAN imaging screen: mean Ca^{2+} response to CS+ and CS- odors on first acquisition trial. Each grey dots represents data from a single fly. Sample sizes as in **Fig. 1d**: n = 8, 9, 8, 8, 11, 8, 9, 11, 9, 11, 8, 9, 8, 8, 10, 9, 7 flies per genotype. Description of violin plots as in **Fig. 1g**.

(**c**) DAN imaging screen: mean Ca^{2+} response of indicated PAM DANs to CS+ and CS- odors on first reversal trial. Sample sizes as in (**b**).

(**d**) Neural activity of PAM-β′2a in flies undergoing aversive memory acquisition and reversal trials, using reciprocal odor identities, i.e., 3-octanol (OCT) and 4-methyl-cyclohexanol (MCH) as CS+ and CS- odors respectively. PAM-β′2a CS+ odor response decreases during acquisition and increases during reversal. n = 19 flies. Statistical comparisons as in **Fig. 1g**. Sample fluorescent image is at 20x, scale bar is 20 μm.

(**e**) Same as in (**d**), but from flies undergoing mock acquisition and reversal, i.e., odors are presented as in (**d**), but no shocks are delivered. No change in PAM-β′2a odor response occurs during acquisition or reversal. n = 11 flies. Statistical comparisons as in **Fig. 1g**.

(**f**) Difference in PAM-β′2a mean odor response on 5th versus 1st acquisition trial from experiments in **Fig. 1g, 1h, Supplementary Fig. 1d, 1e**, respectively. n = 9, 11, 19, 11 flies. Error bars are mean ± SEM. A significant decrease in odor response is observed in the CS+ odor in acquisition, compared to mock-acquisition experiments. Statistical comparisons is by unpaired t-test or Wilcoxon matched-pairs signed rank test, n.s. not significant, * p<0.05, ** p<0.01.

Supplementary Figure 2

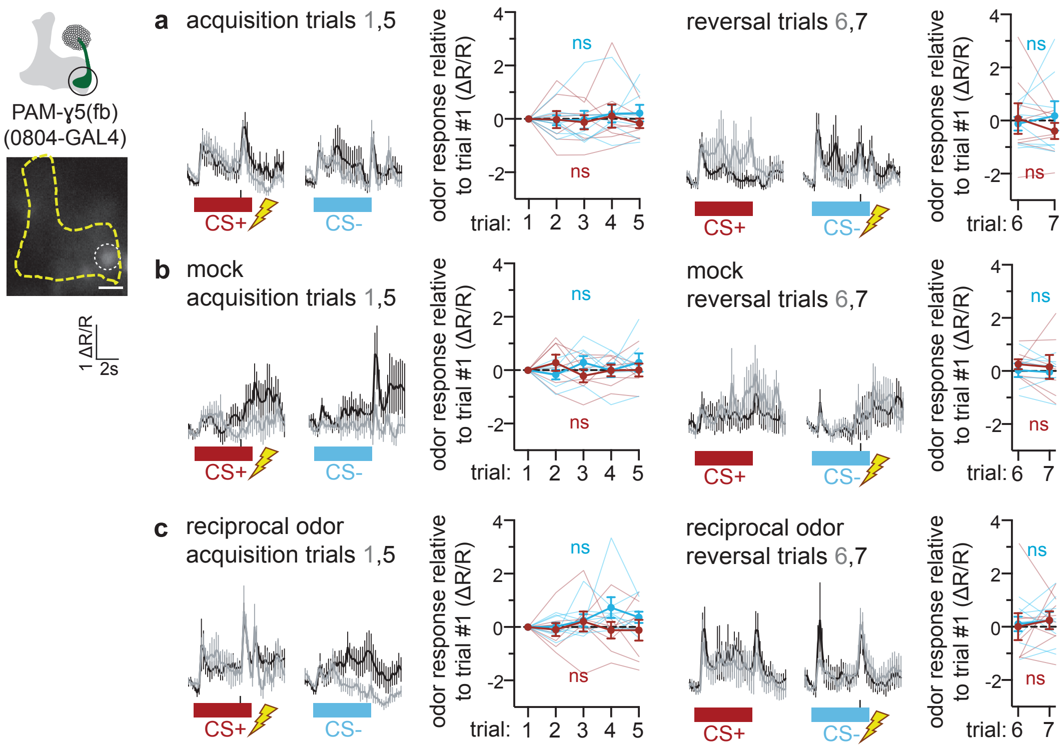

Supplementary Figure 2: Related to Figure 1

(**a**) Neural activity of PAM-γ5(fb) in flies undergoing aversive memory acquisition and reversal, using MCH as CS+. No changes in odor response are observed. $n = 8$ flies. Statistical comparisons as in **Fig. 1g**. Sample fluorescent image is at 20x, scale bar is 20 μm.

(**b**) Neural activity of PAM-γ5(fb) in flies undergoing mock aversive memory acquisition and reversal, using MCH as CS+. No changes in odor response are observed. $n = 7$ flies. Statistical comparisons as in **Fig. 1g**.

(**c**) Neural activity of PAM-γ5(fb) in flies undergoing aversive memory acquisition and reversal, using OCT as CS+. No changes in odor response are observed. $n = 8$ flies. Error bars are mean ± SEM. Statistical comparisons as in **Fig. 1g**, n.s. not significant.

Supplementary Figure 3

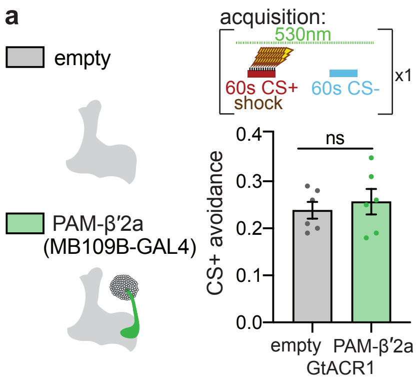

Supplementary Figure 3: Related to Figure 2

(**a**) Above: Experimental protocol for assessing necessity of neurons during aversive memory acquisition using green-light neural silencing via GtACR1. Flies undergo aversive memory acquisition protocol in which 1min of CS+ is paired with 12 electric shocks; 1min of CS- is presented without reinforcement. The shock tube is illuminated with green light throughout acquisition. Below: Control flies acquire a robust aversive memory associated with the CS+ odor and avoid CS+ quadrants. Aversive memory acquisition is not impaired in flies expressing GtACR1 in PAM-β′2a. *n* = 6 independent groups of flies per genotype. Error bars are mean ± SEM. Statistical comparison is by two-tailed unpaired *t*-test, n.s. not significant.

Supplementary Figure 4

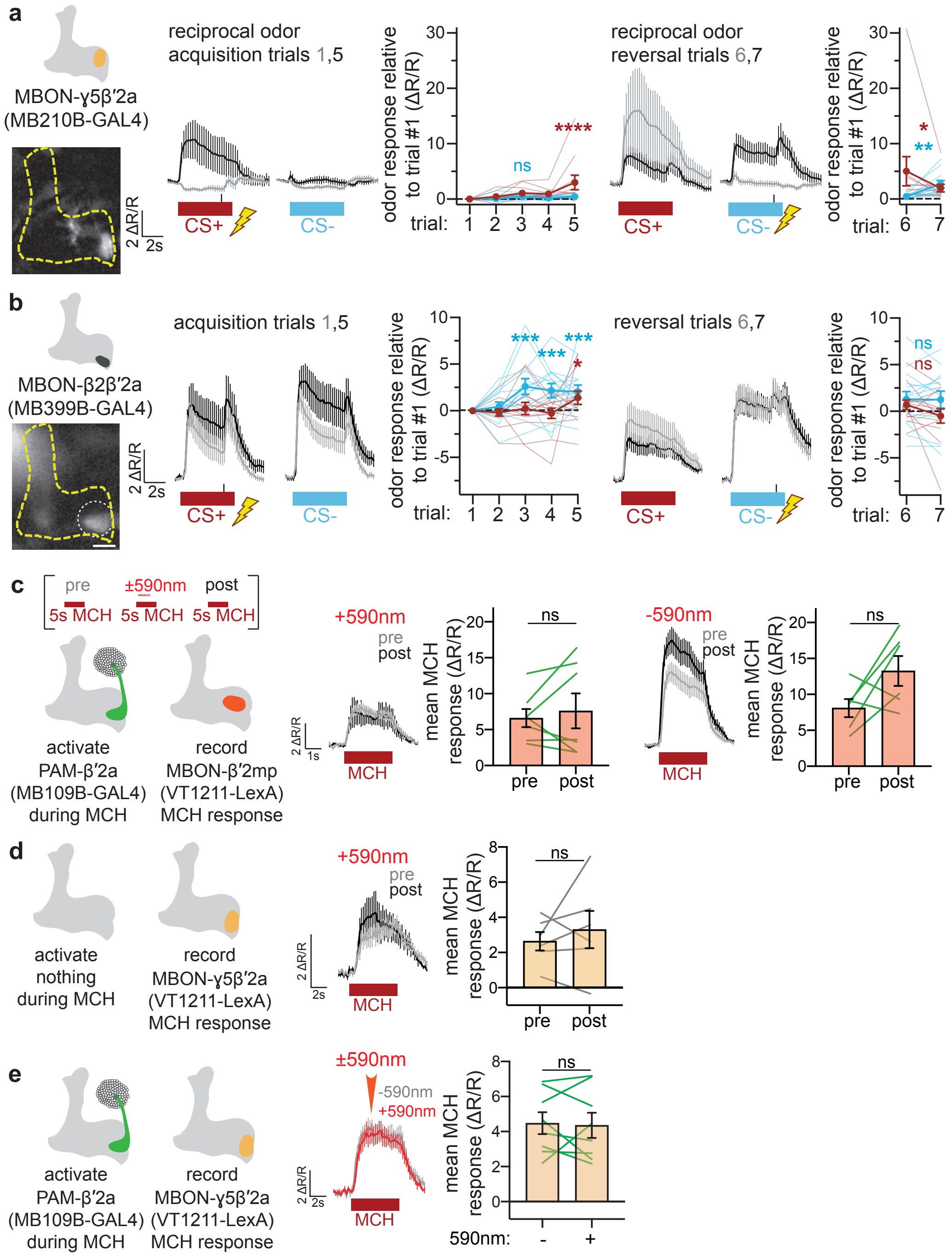

Supplementary Figure 4: Related to Figure 3

(**a**) Neural activity of MBON-γ5β′2a in flies undergoing aversive memory acquisition and reversal, using reciprocal odor identities, i.e. OCT as CS+. MBON-γ5β′2a CS+ odor response increases during acquisition and decreases during reversal. $n = 11$ flies. Statistical comparison as in **Fig. 1g**. Sample fluorescent image is at 20x, scale bar is 20 μm.

(**b**) Neural activity of MBON-β2β′2a in flies undergoing aversive memory acquisition and reversal trials. Both CS+ and CS- odor responses increase in MBON-β2β′2a during acquisition. Importantly, no changes in odor response occur during reversal, making it an unlikely candidate as the postsynaptic partner of PAM-β′2a that decreases CS+ avoidance behavior. $n = 14$ flies. Statistical comparison as in **Fig. 1g**. Sample fluorescent image is at 20x, scale bar is 20 μm.

(**c**) Same experiment as in **Fig. 3f**. Neural responses in MBON-β′2mp before (grey) and after (black) odor-light pairing. Optogenetic activation of PAM-β′2a during MCH odor delivery does not affect subsequent MBON-β′2mp MCH odor response. This is also true in the mock condition. $n = 7$ & 6 flies. Statistical comparison is by paired *t*-test.

(**d**) Same experiment as above, except without expressing Chrimson in any neurons. Odor responses in MBON-γ5β′2a before (grey) and after (black) odor-light pairing are not significantly different. $n = 6$ flies. Statistical comparison is by paired *t*-test.

(**e**) Response in MBON-γ5β′2a during paired odor and PAM-β′2a activation. 5s odor pulse (MCH) is presented to naïve flies, then presented again 2 minutes later with a 500ms 590nm red light pulse that occurs 2s after odor onset. MBON-γ5β′2a neural responses, specifically the mean activity during the 2s after red light offset, i.e., $t = 2.5$-4.5s after odor onset, are compared from each trial. No significant differences between MBON-γ5β′2a neural responses with or without PAM-β′2a activation are observed. $n = 8$ flies. Error bars are mean ± SEM. Statistical comparison is by paired *t*-test, n.s. not significant, * $p<0.05$, ** $p<0.01$, *** $p<0.001$, **** $p<0.0001$.

Supplementary Figure 5

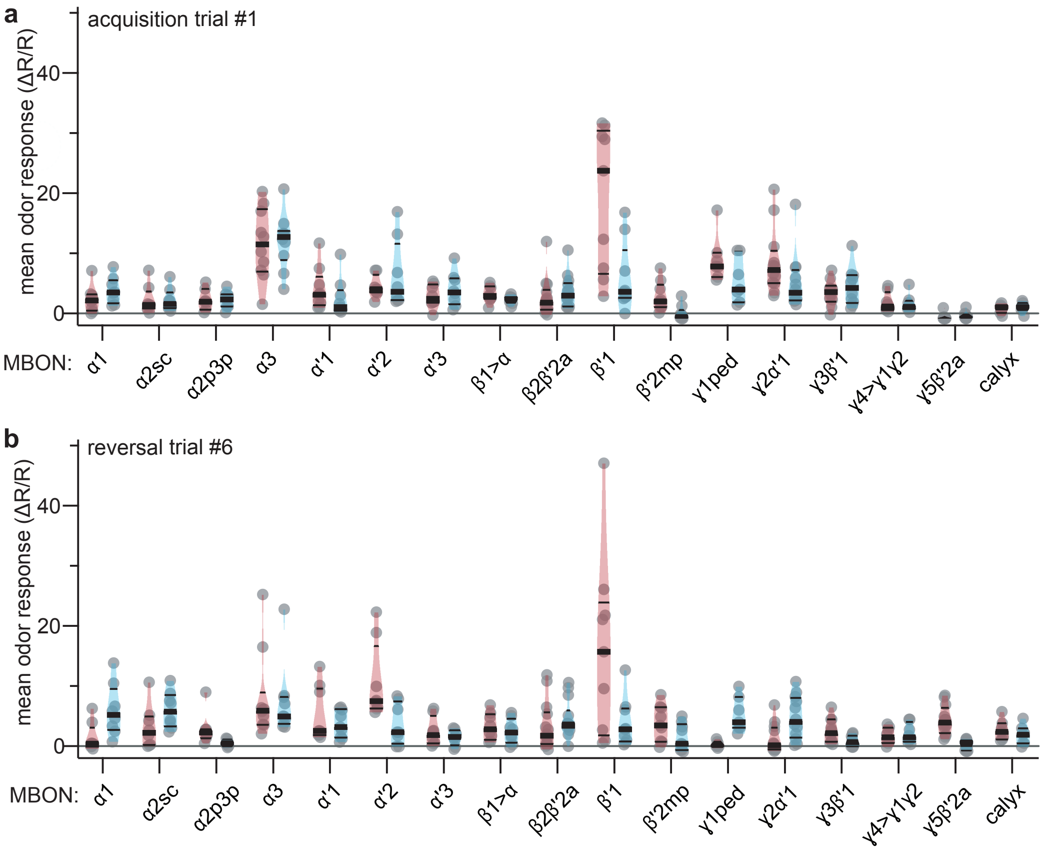

Supplementary Figure 5: Related to Figure 4

(**a**) MBON imaging screen: mean Ca^{2+} response of indicated PAM DANs to CS+ and CS- odors on first acquisition trial. Each grey dots represents data from a single fly. Violin plots show range of data points, with median (thick black line) and quartiles (thin black lines). Sample sizes as in **Fig. 4a**: *n* = 8, 8, 7, 10, 9, 8, 8, 8, 14, 9, 8, 7, 12, 10, 8, 12, 7 flies per genotype.

(**b**) MBON imaging screen: mean Ca^{2+} response of indicated PAM DANs to CS+ and CS- odors on first reversal trial. Sample sizes as in (**a**).

Supplementary Figure 6

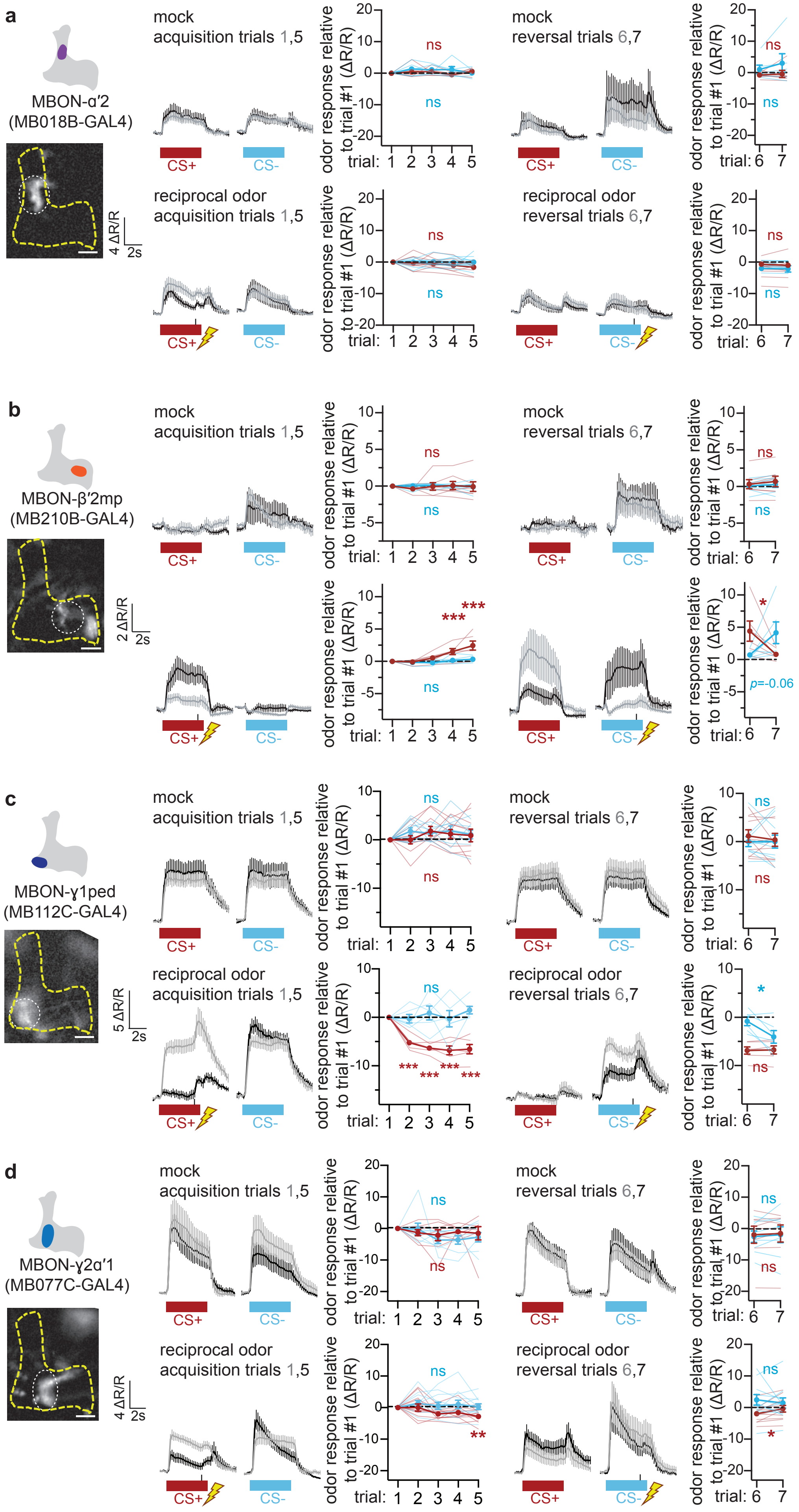

Supplementary Figure 6: Related to Figure 4

(**a**) No change in MBON-α′2 odor response were observed during mock acquisition or reversal, or acquisition or reversal using reciprocal odors (i.e., 3-octanol and 4-methylcyclohexanol as CS+ and CS- respectively). *n* = 6 & 10 flies. Statistical comparison as in **Fig. 1g**.

(**b**) No change in MBON-β′2mp odor response occurs during mock acquisition or reversal. CS+ odor response increases during acquisition, and CS odor response decreases during reversal using reciprocal odors *n* = 7 & 6 flies. Statistical comparison as in **Fig. 1g**.

(**c**) No change in MBON-γ1ped odor response occurs during mock acquisition or reversal. CS+ odor response decreases during acquisition, and CS- odor response decreases during reversal using reciprocal odors. *n* = 10 & 6 flies. Statistical comparison as in **Fig. 1g**.

(**d**) No change in MBON-γ2α′1 odor response occurs during mock acquisition or reversal. CS+ odor response decreases during acquisition, and increases during reversal using reciprocal odors. *n* = 8 & 11 flies. Error bars are mean ± SEM. Statistical comparison as in **Fig. 1g**, n.s. not significant, * $p<0.05$, ** $p<0.01$, *** $p<0.001$. All sample fluorescent images in this figure are at 20x, scale bars are 20 μm.

Supplementary Figure 7

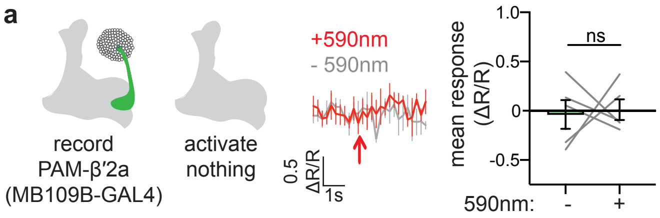

Supplementary Figure 7: Related to Figure 5

(**a**) Same experiment as in **Fig. 5g,h**, except without expressing Chrimson in any neurons. PAM-β′2a activity does not change during red light illumination. *n* = 5 flies. Error bars are mean ± SEM. Statistical comparison is by two-tailed paired *t*-test.

Supplementary Figure 8

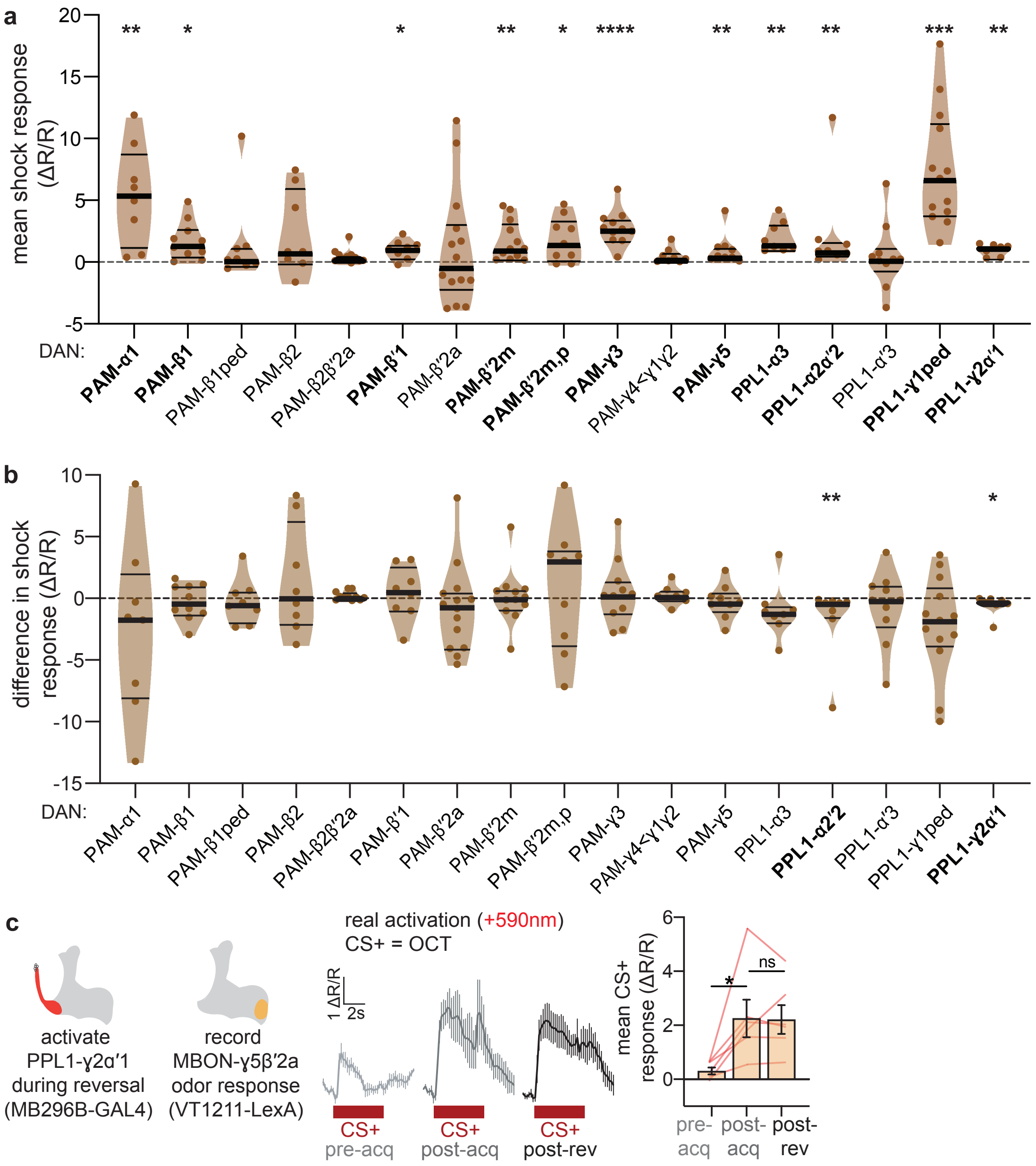

Supplementary Figure 8: Related to Figure 6

(**a**) Mean shock response of each DAN subset during aversive memory acquisition portion of imaging screen. Mean shock response is defined as average activity starting 800ms from electric shock onset, averaged across all 5 acquisition trials. Since CS+ odor offset occurs 1s after shock onset, the mean shock response is defined as such to avoid the time window during odor offset to prevent confounds. Violin plots show range of data points, with median (thick black line) and quartiles (thin black lines). Most PPL1 DANs respond to electric shock, as expected. Surprisingly, many PAM DANs also respond to electric shock. Sample sizes as in **Fig. 1d**: n = 8, 9, 8, 8, 11, 8, 9, 11, 9, 11, 8, 9, 8, 8, 10, 9, 7 flies per genotype. Statistical comparison is by uncorrected one-sample *t*-test or Wilcoxon signed-rank test for each genotype.

(**b**) Difference in mean shock response between first and last acquisition trials. Positive values indicate a relative increase in shock response over the course of acquisition. Sample sizes as in (**a**). Statistical comparison is by uncorrected one-sample *t*-test or Wilcoxon signed-rank test for each genotype.

(**c**) Same experiment as in **Fig. 6b-e**, using reciprocal odors. Optogenetic activation of PPL1-γ2α′1 DAN during CS+ (OCT) presentation in first reversal trial prevents decrease in MBON-γ5β′2a CS+ odor response. n = 6 flies. Error bars are mean ± SEM. Statistical comparison is by one-way ANOVA with Dunnett's post-hoc test against post-acquisition, n.s. not significant, * $p<0.05$, ** $p<0.01$, *** $p<0.001$, **** $p<0.0001$.

Supplementary Figure 9

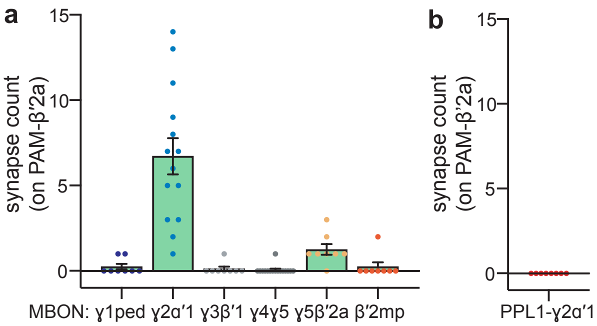

Supplementary Figure 9: Related to Figure 6

(**a**) Number of synapses between each γ lobe MBON and PAM-β′2a. MBON-γ2α′1 synapses plotted again (as in **Fig. 6h**) for ease of comparison. MBON-γ2α′1 has more synaptic connections on PAM-β′2a than any other γ lobe MBON.

(**b**) There are no synaptic connections between PPL1-γ2α′1 and PAM-β′2a.

Supplementary Table 1

<table>
<tr><td>Fly strains</td><td></td><td>Reference</td><td>Source</td></tr>
<tr><td>UAS-GCaMP6f;
UAS-tdTomato</td><td></td><td>Chen et al., 2013</td><td>Gift from D.A Clark</td></tr>
<tr><td>UAS-GtACR1</td><td></td><td>Mohammad et al., 2017</td><td>Gift from A. Claridge-Chang</td></tr>
<tr><td>20xUAS-CsChrimson-mVenus</td><td></td><td>Klapoetke et al., 2014</td><td>BDSC: 55135</td></tr>
<tr><td>20xUAS-GCaMP6m (attp2), 13xLexAop2-CsChrimson-mCherry (vk5)</td><td></td><td>Franconville et al., 2018</td><td>Gift from Janelia FlyLight</td></tr>
<tr><td>20xUAS-CsChrimson-mCherry (attp1), 13xLexAop2-GCaMP6m (vk5)</td><td></td><td>Franconville et al., 2018</td><td>Gift from Janelia FlyLight</td></tr>
<tr><td></td><td></td><td></td><td></td></tr>
<tr><td>spGAL4</td><td>Empty splitGAL4 line</td><td>Hampel et al., 2015</td><td>Flylight: 3019156</td></tr>
<tr><td>R39A05-GAL4</td><td>MBON-β′2mp*</td><td>Jenett et al., 2012</td><td>BDSC: #50033</td></tr>
<tr><td>R66C08-GAL4</td><td>MBON-γ5β′2a*</td><td>Owald et al., 2015</td><td>BDSC: #49412</td></tr>
<tr><td>0804-GAL4</td><td>PAM-γ5(fb)</td><td>Otto et al., 2020</td><td>BDSC: #63825</td></tr>
<tr><td>VT1211-LexA</td><td>MBON-β′2mp & MBON-γ5β′2a</td><td>Felsenberg et al., 2018</td><td>Gift from S. Waddell</td></tr>
<tr><td>R25D01-LexA</td><td>MBON-γ2α′1**</td><td>Jenett et al., 2012</td><td>BDSC: #53519</td></tr>
<tr><td>MB310C-GAL4</td><td>MBON-α1</td><td rowspan="20">Aso et al., 2014a</td><td>Flylight: 2135358</td></tr>
<tr><td>MB080C-GAL4</td><td>MBON-α2sc</td><td>Flylight: 2135128</td></tr>
<tr><td>MB542B-GAL4</td><td>MBON-α2p3p</td><td>Flylight: 2501887</td></tr>
<tr><td>MB082C-GAL4</td><td>MBON-α3</td><td>Flylight: 2135130</td></tr>
<tr><td>MB050B-GAL4</td><td>MBON-α′1</td><td>Flylight: 2135100</td></tr>
<tr><td>MB018B-GAL4</td><td>MBON-α′2</td><td>Flylight: 2135069</td></tr>
<tr><td>MB027B-GAL4</td><td>MBON-α′3</td><td>Flylight: 2135078</td></tr>
<tr><td>MB433B-GAL4</td><td>MBON-β1>α</td><td>Flylight: 2501774</td></tr>
<tr><td>MB399B-GAL4</td><td>MBON-β2β′2a</td><td>Flylight: 2501738</td></tr>
<tr><td>MB057B-GAL4</td><td>MBON-β′1</td><td>Flylight: 2135106</td></tr>
<tr><td>MB210B-GAL4</td><td>MBON-β′2mp & MBON-γ5β′2a</td><td>Flylight: 2135062</td></tr>
<tr><td>MB112C-GAL4</td><td>MBON-γ1ped</td><td>Flylight: 2135160</td></tr>
<tr><td>MB077B-GAL4</td><td>MBON-γ2α′1</td><td>Flylight: 2135209</td></tr>
<tr><td>MB110C-GAL4</td><td>MBON-γ3β′1</td><td>Flylight: 2135158</td></tr>
<tr><td>MB298B-GAL4</td><td>MBON-γ4>γ1γ2</td><td>Flylight: 2135346</td></tr>
<tr><td>MB242A-GAL4</td><td>MBON-calyx</td><td>Flylight: 2135290</td></tr>
<tr><td>MB043C-GAL4</td><td>PAM-α1</td><td>Flylight: 2501609</td></tr>
<tr><td>MB058B-GAL4</td><td>PPL1-α2α′2</td><td>Flylight: 2135107</td></tr>
<tr><td>MB304B-GAL4</td><td>PPL1-α′3</td><td>Flylight: 2135352</td></tr>
<tr><td>MB063B-GAL4</td><td>PAM-β1</td><td>Flylight: 2135111</td></tr>
</table>

MB194B-GAL4	PAM-β1ped	Aso et al., 2014a	Flylight: 2135242
MB213B-GAL4	PAM-β2		Flylight: 2135261
MB301B-GAL4	PAM-β2β′2a		Flylight: 2135349
MB025B-GAL4	PAM-β′1		Flylight: 2135076
MB032B-GAL4	PAM-β′2a		Flylight: 2135083
MB109B-GAL4	PAM-β′2m		Flylight: 2135157
MB047B-GAL4	PAM-β′2m,p		Flylight: 2135097
MB320C-GAL4	PPL1-γ1ped		Flylight: 2135368
MB296B-GAL4	PPL1-γ2α′1		Flylight: 2135344
MB441B-GAL4	PAM-γ3		Flylight: 2501782
MB312B-GAL4	PAM-γ4<γ1γ2		Flylight: 2135089
MB315C-GAL4	PAM-γ5		Flylight: 2135363

* - R39A05-GAL4 and R66C08-GAL4 also have expression in the antennal lobe, antennal mechanosensory and motor center, ellipsoid body, epaulette, noduli, protocerebrum, saddle and optic tubercle.

** - R25D01-LexA also has expression in the bulb, ellipsoid body, epaulette, gall, inferior posterior slope, lateral accessory lobe, noduli, optic tubercle, prow, and protocerebrum.

Statistics for Main Figures

<u>Imaging screen data (difference in CS+ versus CS- odor response)</u>

Normality is determined using D'Agostino & Pearson test; in cases where the *n* is too small, Kolmogorov-Smirnov test is used. two-tailed paired *t*-test (between CS+ and CS-) is used for normal data, and Wilcoxon matched-pairs signed rank test is used for non-normal data. Mean (M) refers to mean difference between CS+ and CS- values.

Fig. 1d,e:

Genotype	Acquisition (Fig. 1d)						Reversal (Fig. 1e)				
	n	Stat. test	M	SD	*t* or *W*	*p*	Stat. test	M	SD	*t* or *W*	*p*
PAM-α1	8	*t*-test	-1.17	3.08	1.08	0.32	Wilcoxon	0.71	1.57	16.00	0.31
PAM-β1	9	*t*-test	0.10	2.58	0.11	0.91	*t*-test	1.18	1.86	1.90	0.09
PAM-β1ped	8	*t*-test	-0.28	5.16	1.80	0.11	*t*-test	0.71	5.72	1.21	0.27
PAM-β2	8	*t*-test	-2.06	4.13	1.41	0.20	*t*-test	0.71	3.48	0.58	0.58
PAM-β2β′2a	11	Wilcoxon	-0.28	2.41	-4.00	0.90	Wilcoxon	0.05	2.52	18.00	0.46
PAM-β′1	8	*t*-test	0.20	4.08	0.14	0.89	*t*-test	-2.09	4.06	1.46	0.19
PAM-β′2a	9	*t*-test	-8.12	7.47	3.08	0.002	Wilcoxon	7.86	9.83	43.00	0.008
PAM-β′2m	11	*t*-test	-2.02	1.74	3.86	0.003	*t*-test	0.23	2.10	0.36	0.73
PAM-β′2m,p	9	Wilcoxon	-8.02	14.55	-33.00	0.05	*t*-test	-0.18	6.80	0.08	0.94
PAM-γ3	11	Wilcoxon	0.68	2.13	34.00	0.15	Wilcoxon	0.04	1.64	18.00	0.46
PAM-γ4<γ1γ2	8	*t*-test	-2.06	3.28	1.78	0.12	*t*-test	0.48	1.37	0.10	0.35
PAM-γ5	9	*t*-test	0.54	1.01	1.62	0.14	*t*-test	-0.09	0.74	0.38	0.72
PPL1-α3	8	*t*-test	1.86	2.58	2.04	0.08	*t*-test	-0.47	0.87	1.51	0.17
PPL1-α2α′2	8	*t*-test	1.21	2.79	1.23	0.26	*t*-test	-0.60	1.64	1.03	0.34
PPL1-α′3	10	Wilcoxon	-0.35	7.10	-21.00	0.32	*t*-test	0.76	9.64	0.25	0.81
PPL1-γ1ped	9	*t*-test	2.01	1.18	5.08	0.0009	*t*-test	-0.44	1.09	1.21	0.26
PPL1-γ2α′1	7	*t*-test	-1.81	2.41	1.98	0.10	*t*-test	-0.19	2.16	0.23	0.82

Fig. 4a,b:

Genotype	Acquisition (Fig. 4a)						Reversal (Fig. 4b)				
	n	Stat. test	M	SD	*t* or *W*	*p*	Stat. test	M	SD	*t* or *W*	*p*
MBON-α1	8	*t*-test	-2.27	4.30	1.50	0.18	*t*-test	3.30	4.28	2.18	0.07
MBON-α2sc	8	*t*-test	-0.61	3.34	0.52	0.62	*t*-test	0.32	3.05	0.30	0.77
MBON-α2p3p	7	*t*-test	0.87	2.97	0.77	0.47	*t*-test	0.04	2.36	0.04	0.97
MBON-α3	10	*t*-test	2.13	8.23	0.82	0.43	*t*-test	1.09	4.68	0.74	0.48
MBON-α′1	9	Wilcoxon	-0.88	5.05	-17.00	0.36	*t*-test	0.65	2.15	0.91	0.39
MBON-α′2	8	*t*-test	6.48	9.79	1.87	0.10	Wilcoxon	-3.28	4.90	-32.00	0.02
MBON-α′3	8	*t*-test	1.57	3.19	1.39	0.21	*t*-test	1.48	2.73	1.54	0.17
MBON-β1>α	8	*t*-test	-0.44	1.70	0.74	0.49	*t*-test	-0.60	2.82	0.60	0.56
MBON-β2β′2a	14	*t*-test	-0.71	2.55	1.04	0.32	*t*-test	-1.14	3.17	1.35	0.20
MBON-β′1	9	*t*-test	1.41	17.04	0.25	0.81	*t*-test	-1.16	8.85	0.39	0.70
MBON-β′2mp	8	*t*-test	2.30	3.62	1.80	0.12	*t*-test	-2.87	3.62	2.38	0.04
MBON-γ1ped	7	*t*-test	-7.86	4.54	4.58	0.004	*t*-test	2.71	2.70	3.01	0.02
MBON-γ2α′1	12	Wilcoxon	-5.05	9.48	-48.00	0.06	Wilcoxon	4.03	6.48	60.00	0.02
MBON-γ3β′1	10	*t*-test	2.55	4.11	1.97	0.08	*t*-test	-0.56	2.10	0.84	0.42
MBON-γ4>γ1γ2	8	*t*-test	0.17	1.89	0.25	0.81	*t*-test	0.17	1.34	0.35	0.74
MBON-γ5β′2a	12	Wilcoxon	-7.86	4.54	76	0.001	*t*-test	-3.46	3.27	3.66	0.004
MBON-calyx	7	*t*-test	0.73	1.79	1.07	0.32	*t*-test	-0.31	3.13	0.26	0.80

Imaging data for individual genotypes (odor response during acquisition)

Imaging data for individual genotypes during acquisition were analyzed using repeated-measures two-way ANOVA, with odor (CS+ vs CS-) and trial # (1-5) as factors. *F* values for odor (*df* = 1), trial number (*df* = 4), and interaction (“int”, *df* = 4) are provided.

Fig. 1d

DAN	*n*	*F* (odor)	*p* (odor)	*F* (trial)	*p* (trial)	*F* (int)	*p* (int)
PAM-α1	8	4.00	0.09	1.19	0.34	0.79	0.54
PAM-β1	9	19.8	0.002	2.22	0.09	1.05	0.39
PAM-β1ped	8	21.6	0.002	0.68	0.061	1.24	0.32
PAM-β2	8	5.88	0.05	5.29	0.003	1.84	0.15
PAM-β2β′2a	11	33.9	0.0002	1.74	0.16	1.85	0.14
PAM-β′1	8	2.41	0.16	3.59	0.02	0.17	0.95
PAM-β′2a	9	12.1	0.008	2.14	0.10	3.14	0.03
PAM-β′2m	11	22.9	0.0007	4.95	0.003	5.56	0.001
PAM-β′2m,p	9	1.12	0.32	1.33	0.28	2.31	0.08
PAM-γ3	11	1.06	0.33	0.77	0.55	0.86	0.50
PAM-γ4<γ1γ2	8	8.13	0.02	6.74	0.0006	2.57	0.06
PAM-γ5	9	3.80	0.09	0.14	0.97	1.50	0.23
PPL1-α3	8	6.04	0.04	6.15	0.001	2.07	0.11
PPL1-α2α′2	8	35.5	0.0006	1.78	0.16	0.97	0.44
PPL1-α′3	10	0.03	0.86	2.27	0.08	0.76	0.56
PPL1-γ1ped	9	9.66	0.01	0.19	0.94	3.42	0.02
PPL1-γ2α′1	7	21.7	0.004	4.18	0.01	2.03	0.12

Fig. 4a

MBON	*n*	*F* (odor)	*p* (odor)	*F* (trial)	*p* (trial)	*F* (int)	*p* (int)
MBON-α1	8	34.5	0.0006	0.74	0.57	0.69	0.60
MBON-α2sc	8	0.29	0.60	4.78	0.005	0.27	0.90
MBON-α2p3p	7	0.22	0.66	2.95	0.04	2.19	0.10
MBON-α3	10	1.33	0.28	3.50	0.02	0.97	0.44
MBON-α′1	9	3.63	0.09	0.42	0.79	0.75	0.57
MBON-α′2	8	13.7	0.008	1.71	0.18	2.67	0.05
MBON-α′3	8	0.20	0.67	1.90	0.14	2.80	0.05
MBON-β1>α	8	2.45	0.16	1.47	0.24	1.30	0.29
MBON-β2β′2a	14	55.9	<0.0001	2.67	0.04	4.62	0.003
MBON-β′1	9	9.00	0.02	0.49	0.74	0.27	0.90
MBON-β′2mp	8	1.02	0.34	1.93	0.13	3.24	0.02
MBON-γ1ped	7	9.53	0.03	14.8	<0.0001	13.8	<0.0001
MBON-γ2α′1	12	0.06	0.81	1.17	0.34	2.70	0.04
MBON-γ3β′1	10	3.05	0.11	7.49	0.0002	2.51	0.06
MBON-γ4>γ1γ2	8	0.35	0.57	0.78	0.55	0.82	0.53
MBON-γ5β′2a	12	49.0	<0.0001	17.6	<0.0001	15.1	<0.0001
MBON-calyx	7	1.41	0.28	11.0	<0.0001	1.17	0.35

Fig #	Description of acquisition experiment	CS+	*n*	*F* (odor)	*p* (odor)	*F* (trial)	*p* (trial)	*F* (int)	*p* (int)
1g	PAM-β′2a	MCH	9	7.23	0.03	2.62	0.08	4.43	0.02
1h	PAM-β′2a, mock	MCH	11	1.88	0.20	0.37	0.73	0.93	0.42
3b	MBON-γ5β′2a	MCH	12	49.03	<0.0001	17.58	<0.0001	15.07	<0.0001
3c	MBON-γ5β′2a, mock	MCH	8	1.12	0.33	1.09	0.38	0.68	0.61
4c	MBON-α′2	MCH	8	3.44	0.11	1.71	0.18	2.67	0.05
4d	MBON-β′2mp	MCH	9	3.02	0.12	1.93	0.13	3.24	0.02
4e	MBON-γ1ped	MCH	7	21.77	<0.0001	14.82	<0.0001	13.82	<0.0001
4f	MBON-γ2α′1	MCH	12	4.25	0.06	1.80	0.15	2.46	0.05

<u>Imaging data for individual genotypes (odor response during reversal)</u>

As with the screen data, either one sample / paired *t*-test or Wilcoxon matched-pairs signed rank test was used on data that were normal and non-normal, respectively.

Fig. 1e

DAN	*n*	Stat. test (CS+)	*t* or *W* (CS+)	*p* (CS+)	Stat. test (CS-)	*t* or *W* (CS-)	*p* (CS-)
PAM-α1	8	Wilcoxon	0	0.99	*t*-test	1.49	0.18
PAM-β1	9	*t*-test	0.76	0.47	*t*-test	1.09	0.31
PAM-β1ped	8	Wilcoxon	18	0.25	*t*-test	0.79	0.46
PAM-β2	8	*t*-test	0.67	0.52	*t*-test	0.65	0.54
PAM-β2β′2a	11	*t*-test	1.98	0.08	Wilcoxon	-30	0.21
PAM-β′1	8	*t*-test	1.07	0.32	*t*-test	0.91	0.39
PAM-β′2a	9	*t*-test	2.60	0.03	Wilcoxon	-41	0.01
PAM-β′2m	11	*t*-test	0.10	0.92	*t*-test	0.24	0.81
PAM-β′2m,p	9	*t*-test	0.32	0.76	Wilcoxon	-9	0.65
PAM-γ3	11	Wilcoxon	24	0.32	*t*-test	0.43	0.68
PAM-γ4<γ1γ2	8	*t*-test	0.24	0.82	*t*-test	1.98	0.09
PAM-γ5	9	*t*-test	0.39	0.71	*t*-test	0.26	0.80
PPL1-α3	8	*t*-test	1.78	0.12	Wilcoxon	20	0.20
PPL1-α2α′2	8	*t*-test	0.58	0.58	*t*-test	0.74	0.48
PPL1-α′3	10	*t*-test	0.69	0.69	*t*-test	0.04	0.97
PPL1-γ1ped	9	*t*-test	0.53	0.53	*t*-test	0.51	0.62
PPL1-γ2α′1	7	*t*-test	0.61	0.61	*t*-test	0.44	0.68

Fig. 4b

MBON	*n*	Stat. test (CS+)	*t* or *W* (CS+)	*p* (CS+)	Stat. test (CS-)	*t* or *W* (CS-)	*p* (CS-)
MBON-α1	8	Wilcoxon	10	0.55	Wilcoxon	-26	0.08
MBON-α2sc	8	*t*-test	0.86	0.42	*t*-test	0.18	0.86
MBON-α2p3p	7	*t*-test	0.99	0.36	*t*-test	2.22	0.07
MBON-α3	10	*t*-test	0.26	0.80	Wilcoxon	-15	0.49
MBON-α′1	9	*t*-test	1.34	0.22	Wilcoxon	-5	0.82
MBON-α′2	8	Wilcoxon	-36	0.008	*t*-test	2.24	0.06
MBON-α′3	8	*t*-test	1.13	0.30	*t*-test	0.91	0.39

MBON-β1>α	8	*t*-test	0.59	0.57	*t*-test	0.08	0.94
MBON-β2β′2a	14	Wilcoxon	-45	0.17	*t*-test	0.10	0.93
MBON-β′1	9	*t*-test	0.59	0.57	*t*-test	0.08	0.94
MBON-β′2mp	8	Wilcoxon	-41	0.01	*t*-test	1.02	0.34
MBON-γ1ped	7	*t*-test	1.16	0.29	*t*-test	3.03	0.02
MBON-γ2α′1	12	Wilcoxon	56	0.03	*t*-test	1.34	0.21
MBON-γ3β′1	10	*t*-test	2.43	0.04	*t*-test	1.79	0.11
MBON-γ4>γ1γ2	8	*t*-test	0.27	0.80	*t*-test	0.68	0.52
MBON-γ5β′2a	12	*t*-test	3.10	0.01	*t*-test	3.42	0.006
MBON-calyx	7	*t*-test	0.29	0.78	*t*-test	0.03	0.98

Fig #	Description of reversal experiment	CS+	*n*	Stat. test	*t* or *W* (CS+)	*p* (CS+)	Stat. test	*t* or *W* (CS-)	*p* (CS-)
1g	PAM-β′2a	MCH	9	*t*-test	2.60	0.03	Wilcoxon	41.00	0.01
1h	PAM-β′2a, mock	MCH	11	*t*-test	1.45	0.17	Wilcoxon	10.00	0.73
3b	MBON-γ5β′2a	MCH	12	*t*-test	3.10	0.01	*t*-test	3.42	0.006
3c	MBON-γ5β′2a, mock	MCH	8	*t*-test	0.39	0.71	*t*-test	1.03	0.34
4c	MBON-α′2	MCH	8	Wilcoxon	36.00	0.008	*t*-test	2.24	0.06
4d	MBON-β′2mp	MCH	9	Wilcoxon	41.00	0.01	*t*-test	1.02	0.34
4e	MBON-γ1ped	MCH	7	*t*-test	1.16	0.29	*t*-test	3.03	0.02
4f	MBON-γ2α′1	MCH	12	Wilcoxon	56.00	0.03	*t*-test	1.34	0.21

Behavioral and functional connectivity data in main figures

Fig #	Description of experiment	Statistic	*n*	*t* or *F*	*p*
1c	Wild-type CS+ avoidance, acq vs rev	Unpaired *t*-test	4, 3	$t = 4.144$	0.009
2b	Wild-type CS+ avoidance, # reversals, CS+ vs CS-	One-way ANOVA + Dunnett's	6, 11, 8	$F = 54.81$	<0.0001
2c	Wild-type CS+ avoidance, # reversals, CS+ vs BA	One-way ANOVA + Dunnett's	2, 5, 5	$F = 16.00$	0.0011
2d	PAM-β′2a activation during odor	Unpaired *t*-test	9, 9	$t = 3.14$	0.0064
2e	PAM-β′2a inhibition during odor	Unpaired *t*-test	9, 11	$t = 2.87$	0.0101
2f	PAM-β′2a activation during reversal	Unpaired *t*-test	8, 8	$t = 2.32$	0.04
2g	PAM-β′2a inhibition during reversal	Unpaired *t*-test	10, 10	$t = 2.78$	0.013
3d	MBON-γ5β′2a activation during reversal	Unpaired *t*-test	8, 7	$t = 2.57$	0.02
3e	MBON-γ5β′2a inhibition during reversal	Unpaired *t*-test	10, 9	$t = 3.14$	0.006
3g	MBON-γ5β′2a odor response, pre-post, +590nm	Paired *t*-test	6	$t = 3.54$	0.02
3h	MBON-γ5β′2a odor response, pre-post, -590nm	Paired *t*-test	6	$t = 0.55$	0.61
5b	MBON-γ2α′1, MBON-β′2mp activation during reversal	One-way ANOVA + Dunnett's	50-50: 12, 8, 7<br>100-50: 11, 10, 12<br>100-100: 12, 9, 7	$F = 4.551$<br>$F = 1.04$<br>$F = 5.44$	0.021<br>0.37<br>0.011

5c	MBON-γ2α′1, MBON-β′2mp inhibition during reversal	One-way ANOVA + Dunnett's	50-50: 16, 6, 8<br>100-50: 22, 12, 10<br>100-100: 15, 14, 9	$F = 11.66$<br>$F = 3.67$<br>$F = 1.82$	0.0002<br>0.034<br>0.18
5e	MBON-γ2α′1, MBON-β′2mp innate red light preference	Mixed-model two-way ANOVA	1: 11, 7, 10<br>5: 6, 7, 7<br>10: 7, 7, 7<br>20: 12, 7, 11<br>50: 5, 5	F(genotype) = 55.55<br>F(intensity) = 13.42<br>F(interaction) = 26.16	<0.0001<br><0.0001<br><0.0001
5g	PAM-β′2a response after MBON-γ2α′1 stimulation, ±590nm	Paired *t*-test	9	$t = 3.49$	0.008
5h	PAM-β′2a response after MBON-γ5β′2a + MBON- β′2mp stimulation, ±590nm	Wilcoxon signed rank test	7	$W = 22.00$	0.08
6a	PAM-γ5, PAM-β′2m, PAM-β′2mp, PPL1-γ2α′1 activation	One-way ANOVA	10, 8, 4, 7, 5	$F = 3.13$	0.03
6d	MBON-γ5β′2a odor response pre-acq, post-acq, post-rev (-590nm)	Repeated-measures one-way ANOVA	6	$F = 6.15$	0.018
6e	MBON-γ5β′2a odor response pre-acq, post-acq, post-rev (+590nm)	Repeated-measures one-way ANOVA	6	$F = 9.36$	0.005

Statistics for Supplementary Figures

Imaging data for individual genotypes (odor response during acquisition)

Fig #	Description of experiment	CS+	*n*	*F* (odor)	*p* (odor)	*F* (trial)	*p* (trial)	*F* (int)	*p* (int)
S1d	PAM-β′2a, reciprocal odor	OCT	19	0.46	0.51	4.69	0.007	2.34	0.10
S1e	PAM-β′2a, mock reciprocal	OCT	11	0.08	0.33	1.19	0.33	0.12	0.98
S2a	PAM-γ5n	MCH	8	0.58	0.47	0.26	0.90	0.62	0.65
S2b	PAM-γ5n, mock	MCH	7	0.08	0.79	0.22	0.93	1.76	0.17
S2c	PAM-γ5n, reciprocal odor	OCT	8	1.12	0.33	0.89	0.49	1.34	0.28
S4a	MBON-γ5β′2a, reciprocal odor	OCT	11	4.56	0.06	5.01	0.0023	3.01	0.029
S4b	MBON-β2β′2a	MCH	14	12.35	0.004	3.03	0.03	5.29	0.001
S6a	MBON-α′2, mock	MCH	6	1.68	0.25	1.02	0.42	1.17	0.35
S6a	MBON-α′2, reciprocal odor	OCT	10	2.74	0.13	1.65	0.18	1.16	0.34
S6b	MBON-β′2mp, mock	MCH	8	0.05	0.91	0.24	0.91	0.30	0.87
S6b	MBON-β′2mp, reciprocal odor	OCT	6	6.96	0.046	10.12	0.0001	6.96	0.0011
S6c	MBON-γ1ped, mock	MCH	10	0.57	0.47	1.73	0.17	0.92	0.46
S6c	MBON-γ1ped, reciprocal odor	OCT	6	26.62	0.004	6.66	0.001	8.56	0.0003
S6d	MBON-γ2α′1, mock	MCH	8	0.62	0.46	2.03	0.12	0.80	0.54
S6d	MBON-γ2α′1, reciprocal odor	OCT	11	7.57	0.02	1.84	0.14	2.14	0.09

Imaging data for individual genotypes (odor response during reversal)

Fig #	Description of experiment	CS+	*n*	Stat. test	*t* or *W* (CS+)	*p* (CS+)	Stat. test	*t* or *W* (CS-)	*p* (CS-)
S1d	PAM-β′2a, reciprocal odor	OCT	19	*t*-test	2.63	0.02	*t*-test	3.77	0.001
S1e	PAM-β′2a, mock reciprocal	OCT	11	Wilcoxon	20.00	0.41	*t*-test	0.40	0.70
S2a	PAM-γ5n	MCH	8	*t*-test	1.28	0.24	*t*-test	0.73	0.49
S2b	PAM-γ5n, mock	MCH	7	*t*-test	0.29	0.78	*t*-test	0.43	0.68
S2c	PAM-γ5n, reciprocal odor	OCT	8	*t*-test	0.47	0.65	*t*-test	0.39	0.71
S4a	MBON-γ5β′2a, reciprocal odor	OCT	11	*t*-test	1.53	0.16	*t*-test	3.71	0.004
S4b	MBON-β2β′2a	MCH	14	Wilcoxon	-43.00	0.19	*t*-test	0.11	0.91
S6a	MBON-α′2, mock	MCH	6	*t*-test	0.33	0.75	*t*-test	1.23	0.27
S6a	MBON-α′2, reciprocal odor	OCT	10	*t*-test	0.36	0.73	*t*-test	1.07	0.31
S6b	MBON-β′2mp mock	MCH	8	*t*-test	0.18	0.86	Wilcoxon	18.00	0.25
S6b	MBON-β′2mp, reciprocal odor	OCT	6	*t*-test	2.78	0.04	*t*-test	2.23	0.08
S6c	MBON-γ1ped, mock	MCH	10	*t*-test	0.75	0.47	*t*-test	0.02	0.98
S6c	MBON-γ1ped, reciprocal odor	OCT	6	*t*-test	0.76	0.48	*t*-test	3.07	0.03
S6d	MBON-γ2α′1, mock	MCH	8	*t*-test	0.68	0.52	*t*-test	0.57	0.58
S6d	MBON-γ2α′1, reciprocal odor	OCT	11	*t*-test	2.92	0.02	*t*-test	0.73	0.48

Behavioral and functional connectivity data

Fig #	Description of experiment	Statistic	*n*	*t* or *F*	*p*
S1a	Train under microscope, single fly	Unpaired *t*-test	11, 11	$t = 3.15$	0.0051
S3a	PAM-β′2a silencing	Unpaired *t*-test	6, 6	$t = 0.57$	0.58
S4c	MBON-β′2mp odor response, pre-post, +590nm	Paired *t*-test	7	$t = 0.37$	0.72
S4c	MBON-β′2mp odor response, pre-post, -590nm	Paired *t*-test	6	$t = 1.99$	0.10
S4d	MBON-γ5β′2a odor response, pre-post, +590nm (no activation)	Paired *t*-test	6	$t = 0.76$	0.48

S4e	MBON-γ5β′2a odor response during PAM-β′2a activation, ±590nm	Paired *t*-test	8	$t = 0.23$	0.82
S7a	PAM-β′2a response after mock stimulation, ±590nm	Paired *t*-test	5	$t = 0.20$	0.85
S8c	MBON-γ5β′2a odor response, reciprocal odor, pre-acq, post-acq, post-rev (+590nm)	Repeated-measures one-way ANOVA	6	$F = 9.53$	0.014

Imaging screen data (shock response during acquisition, Supplementary Fig. 8a,b)

Genotype	Mean (Supp Fig. S8a)				Mean diff (Supp Fig. S8b)		
	n	Stat. test	*t* or *W*	*p*	Stat. test	*t* or *W*	*p*
PAM-α1	8	*t*-test	3.70	0.008	*t*-test	1.06	0.32
PAM-β1	9	*t*-test	3.22	0.01	*t*-test	-0.38	0.43
PAM-β1ped	8	Wilcoxon	12.00	0.46	*t*-test	-0.36	0.60
PAM-β2	8	*t*-test	1.82	0.11	*t*-test	0.83	0.43
PAM-β2β′2a	11	Wilcoxon	42.00	0.07	*t*-test	0.85	0.42
PAM-β′1	8	*t*-test	3.08	0.02	*t*-test	0.42	0.69
PAM-β′2a	9	*t*-test	0.73	0.48	Wilcoxon	-43.00	0.19
PAM-β′2m	11	*t*-test	3.31	0.007	Wilcoxon	-6.00	0.85
PAM-β′2m,p	9	*t*-test	2.78	0.02	*t*-test	0.48	0.65
PAM-γ3	11	*t*-test	6.23	<0.0001	*t*-test	0.44	0.67
PAM-γ4<γ1γ2	8	Wilcoxon	20.00	0.20	*t*-test	0.58	0.58
PAM-γ5	9	Wilcoxon	43.00	0.008	*t*-test	0.78	0.46
PPL1-α3	8	*t*-test	4.37	0.003	Wilcoxon	-22.00	0.15
PPL1-α2α′2	8	Wilcoxon	36.00	0.008	Wilcoxon	-36.00	0.008
PPL1-α′3	10	*t*-test	0.40	0.70	*t*-test	0.86	0.41
PPL1-γ1ped	9	*t*-test	5.59	0.0001	*t*-test	2.07	0.06
PPL1-γ2α′1	7	*t*-test	4.51	0.004	*t*-test	2.17	0.07
