# Supplementary material for: Dopaminergic mechanism underlying reward-encoding of punishment omission during reversal learning in Drosophila
Source: Nat Commun. 2021 Feb 18;12:1115. doi: 10.1038/s41467-021-21388-w (PMC7893153; doi:10.1038/s41467-021-21388-w)
Supplement: Supplementary file 2 — Description of Additional Supplementary files [file 41467_2021_21388_MOESM2_ESM.docx]

**Description of Additional Supplementary Files**

File Name: Supplementary Software 1

Description: Code for analyzing raw GCaMP and tdTomato fluorescence data. This code calculates ratiometric fluorescence values by dividing GCaMP signal by tdTomato signal, processes the resultant trace with a double-exponential fitting to compensate for photobleaching, and converts the resultant trace into ΔR/R0 by normalizing to baseline intensity.
