# Supplementary material for: Dopaminergic mechanism underlying reward-encoding of punishment omission during reversal learning in Drosophila
Source: Nat Commun. 2021 Feb 18;12:1115. doi: 10.1038/s41467-021-21388-w (PMC7893153; doi:10.1038/s41467-021-21388-w)
Supplement: Supplementary file 3 — Supplementary Software [file 41467_2021_21388_MOESM3_ESM.zip › McCurdy et al. Supplementary Software/imaging analysis readme.rtfd/TXT.rtf]

*Please contact liyanmccurdy@gmail.com if you have any questions.*Operating instructionsThis code has been used on Matlab 2017a, on a Mac 10.15.7.What’s in this file: 2 raw data files in excel: 	MB112C_cs+mch.xlsx (MBON-g1ped undergoing acq and rev)	MB112C_cs+mch_mock.xlsx (MBON-g1ped undergoing mock acq and rev)matlab code to analyze the data in the excel files: 	imaginganalysis_7trials.m output of code: .pdf plots of averaged neural traces. How is the raw data generated and organized?Each excel file is for a particular genotype and condition. In this case, recording from MBON-g1ped (MB112C) during acquisition and reversal, CS+ = MCH. Both the actual conditioning and the mock conditioning files are included.There are two sheets in the excel file, one for each odor (CS+ and CS-). Each pair of columns is data from one trial (aka one 20s recording). Data from each fly takes up 12x2=24 columns, so the code is written to accommodate up to 12 trials. (If not providing data for all 12 trials, pad columns with zeros.) Data from each pair of columns comes from Zen software, in which intensity values for each ROI are calculated over time, one column for GCaMP, one column for Tomato. In the excel file, odd columns are GCaMP intensity values, and even columns are Tomato intensity values. Each row is a time point, 200s in this case. So in this sample dataset, there are 7 trials of CS+ and 7 CS-, where 5 are acquisition, and 2 are reversal. For each trial, odor onset begins at t=5s, shock onset at r=9s, odor offset at t=10s. What does the matlab code do?For each excel sheet, the code outputs: 	1) average odor responses for each trial and odor	2) plots the average neural traces (resultantplots.pdf)	3) creates a .csv file for the neural traces, one per odor and trial #.For each GCaMP + Tomato pair of columns, the code processes the data as follows: - Plots the raw GCaMP and ROI trace in blue and the Tomato trace in orange. Raw intensity value is y-axis, time on x-axis. The GCaMP value is divided by the Tomato value, to form variable ‘G’ (line 64).- Calculates double-exponential curve using ‘fit’ function (line 91), to be subtracted from G (line 98).-  Calculates baseline fluorescence level (R0, line 102), as mean fluorescence during first 5s of recording, before any stimulus occurs. .- Calculates dR/R (variable name ‘dFF_G’, line 104).- Calculates mean odor and shock response dR/R. 
