# Supplementary figures and images for: Dopaminergic mechanism underlying reward-encoding of punishment omission during reversal learning in Drosophila
Source: Nat Commun. 2021 Feb 18;12:1115. doi: 10.1038/s41467-021-21388-w (PMC7893153; doi:10.1038/s41467-021-21388-w)

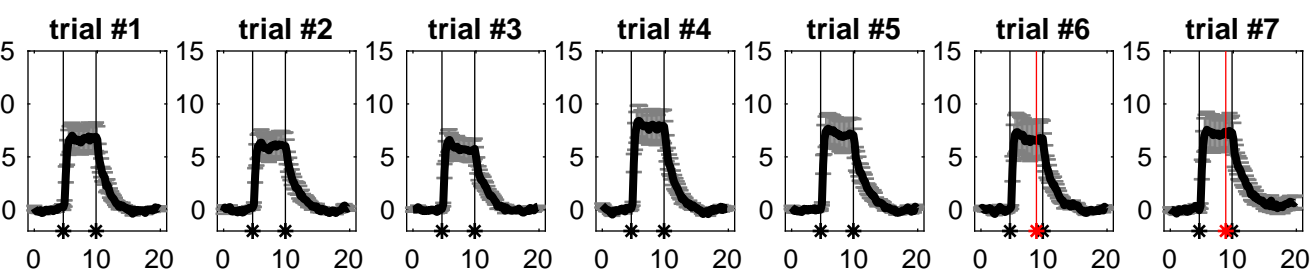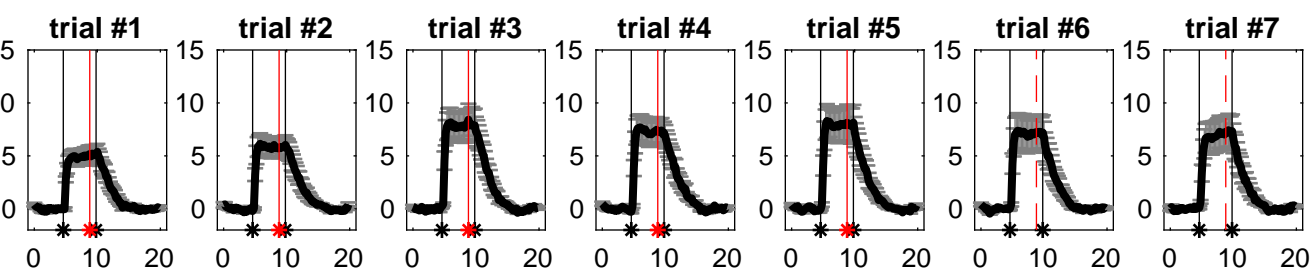

Supplement: Supplementary file 3 — Supplementary Software [file 41467_2021_21388_MOESM3_ESM.zip › McCurdy et al. Supplementary Software/MB112C_cs+mch_mock_plots.pdf]

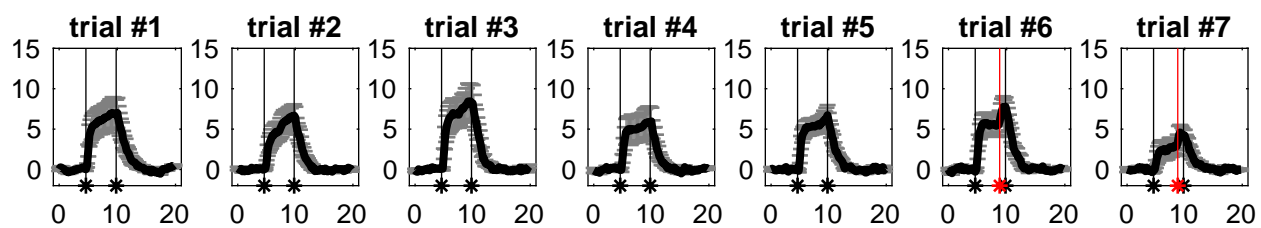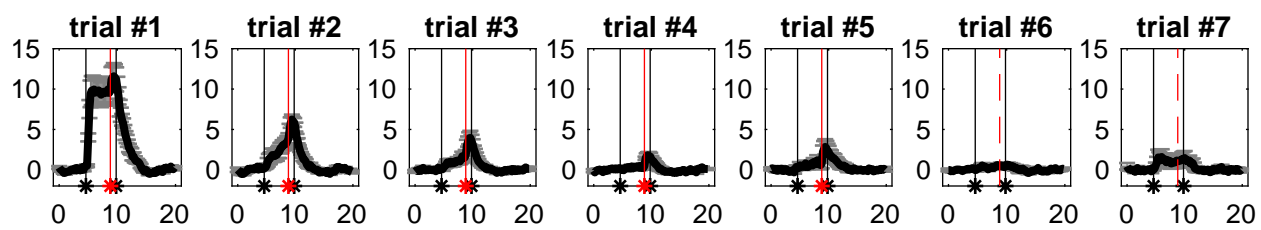

Supplement: Supplementary file 3 — Supplementary Software [file 41467_2021_21388_MOESM3_ESM.zip › McCurdy et al. Supplementary Software/MB112C_cs+mch_plots.pdf]
